# Temperature-Dependent HfO_2_/Si Interface Structural Evolution and its Mechanism

**DOI:** 10.1186/s11671-019-2915-0

**Published:** 2019-03-07

**Authors:** Xiao-Ying Zhang, Chia-Hsun Hsu, Shui-Yang Lien, Wan-Yu Wu, Sin-Liang Ou, Song-Yan Chen, Wei Huang, Wen-Zhang Zhu, Fei-Bing Xiong, Sam Zhang

**Affiliations:** 1 School of Opto-electronic and Communication Engineering, Fujian Provincial Key Laboratory of Optoelectronic Technology and Devices, Xiamen University of Technology, Xiamen, 361024 China; 2 Department of Materials Science and Engineering, Da-Yeh University, ChungHua, 51591 Taiwan; 3 Bachelor Program for Design and Materials for Medical Equipment and Devices, Da-Yeh University, Changhua, 51591 Taiwan; 4 Department of Physics, OSED, Xiamen University, Xiamen, 361005 China; 5 Faculty of Materials and Energy, Southwest University, Chongqing, China

**Keywords:** Hafnium oxide, Atomic layer deposition, Interface, Annealing, Crystallization

## Abstract

In this work, hafnium oxide (HfO_2_) thin films are deposited on p-type Si substrates by remote plasma atomic layer deposition on p-type Si at 250 °C, followed by a rapid thermal annealing in nitrogen. Effect of post-annealing temperature on the crystallization of HfO_2_ films and HfO_2_/Si interfaces is investigated. The crystallization of the HfO_2_ films and HfO_2_/Si interface is studied by field emission transmission electron microscopy, X-ray photoelectron spectroscopy, X-ray diffraction, and atomic force microscopy. The experimental results show that during annealing, the oxygen diffuse from HfO_2_ to Si interface. For annealing temperature below 400 °C, the HfO_2_ film and interfacial layer are amorphous, and the latter consists of HfO_2_ and silicon dioxide (SiO_2_). At annealing temperature of 450-550 °C, the HfO_2_ film become multiphase polycrystalline, and a crystalline SiO_2_ is found at the interface. Finally, at annealing temperature beyond 550 °C, the HfO_2_ film is dominated by single-phase polycrystalline, and the interfacial layer is completely transformed to crystalline SiO_2_.

## Introduction

Hafnium oxide (HfO_2_) thin film is an interesting material for a variety of applications. It can be used in multilayer optical coating [1], protective coating [2], gate dielectric [3], passivating layer [4–6], and so on due to its excellent properties, such as high density, high refractive index, wide band gap, and relatively high thermal stability. Many methods have been used to prepare HfO_2_ thin film, such as electron beam evaporation [7], chemical solution deposition [8], reactive sputtering [9], metal organic chemical vapor deposition [10], molecular beam epitaxy [11], and atomic layer deposition (ALD). ALD is a promising method for obtaining thin films with both high-precision thickness control and high accuracy uniformity. Post-annealing is found to have significant influences on ALD HfO_2_ films [12–15]. According to the research, HfO_2_ thin films can crystalize for an annealing temperature higher than 500 °C [16–18]. The crystalline structure of HfO_2_ strongly affects optical and electrical properties. For example, the structural change of HfO_2_ from amorphous to monoclinic crystalline phase could lead to changes of refractive index from 1.7 to 2.09, optical gap from 5.75 to 6.13 eV, and dielectric constant from 24.5 to 14.49 [19, 20]. For ALD HfO_2_ deposited on silicon substrates, an oxide layer is usually observed at HfO_2_/Si interface [21, 22]. The presence of this interfacial layer is reported to decrease the dielectric constant [22]. In addition, Kopani et al. [23] presented the structural properties of 5-nm HfO_2_ films after nitric acid oxidation of n-doped Si substrates. They found that high annealing temperature increases the growth rate of crystalline nuclei. However, their crystallization properties particularly HfO_2_/substrate interface have scantly been studied. Therefore, the annealing temperature affecting the crystallization properties of HfO_2_ thin films prepared by ALD was worth for further investigation.

In this work, the HfO_2_ thin films were fabricated by a remote plasma atomic layer deposition (RP-ALD) on p-type silicon substrates. Post-annealing was performed by a rapid thermal annealing (RTA) system at different temperatures. The structural changes and crystallization properties of HfO_2_ thin films by RTA were characterized by atomic force microscopy (AFM), grazing incident X-ray diffraction (GIXRD), X-ray photoelectron spectroscopy (XPS), and high-resolution transmission electron microscopy (HR-TEM). The temperature-dependent HfO_2_/Si interface structural evolution and its mechanism are also investigated.

## Method

Doubled-sided polished (100) oriented p-type 2-inch 250-μm Czochralski Si wafers with a resistivity of 30 Ω cm were used. Prior to the deposition, Si wafers were cleaned by a standard Radio Corporation of America method followed by dipping in diluted hydrofluoric acid solution (5%) for 2 min to remove possible stray oxides without final water rinse. After cleaning, all of the wafers were dried with pure nitrogen (N_2_) gas and mounted onto the substrate holder. Approximately 15 nm HfO_2_ (168 ALD cycles) thin films were deposited on Si wafers by RP-ALD (Picosun R-200, Finland) using tetrakis (ethylmethylamino) hafnium (TEMAH) and oxygen (O_2_) in alternating pulse with N_2_ purge of the reaction chamber between pulses. The TEMAH and O_2_ plasma were pulsed into the reactor in the following sequence: TEMAH pulse 1.6 s; N_2_ purge 10 s; O_2_ plasma pulse 10 s, and N_2_ purge 12 s. After depositing the HfO_2_ thin films, the rapid thermal annealing was performed in N_2_ ambient for 10 min. The annealing temperatures were varied from 400 to 600 °C to investigate the effect on crystallization of the HfO_2_ thin films and HfO_2_/Si interface. Table 1 lists the typical conditions of RPALD and post-annealing.

**Table 1:** RPALD HfO_2_ deposition parameters

RPALD- HfO_2_ thin film	
Parameter	Value
Substrate temperature (°C)	250
TEMAH pulse time (s)	1.6
O_2_ plasma pulse time (s)	10
O_2_ plasma power (W)	2500
Thickness (nm)	15
RTA-post annealing process	
Parameter	Value
Temperature (°C)	400–600
Time (min)	20
Ambient	N_2_

AFM measurements were performed in tapping mode for investigating the surface morphology of the HfO_2_ thin films. The AFM images shown in this work are 2 μm × 2 μm scans with a resolution of 256 points × 256 lines. The structure of HfO_2_ films were characterized by grazing incident X-ray diffraction (GIXRD, Rigaku TTRAXIII, Japan) measurements with a Cu long-fine-focus X-ray tube. X-rays with a wavelength of 0.154 nm were produced at an operating voltage of 50 kV and a current of 300 mA. An incident angle of 0.5° was selected to obtain diffraction patterns over a 2*θ* range of 20–60°. X-ray photoelectron spectroscopy (XPS, Thermo Fisher K-alpha) was also performed using monochromatic Al Kα X-ray radiation (hν = 1486.6 eV). For the XPS analysis, a 100-μm diameter spot was used, and photoelectrons were collected at a take-off angle of 45°. The cross sections of the HfO_2_ thin films were prepared by a focused ion beam lift-out technique in a Hitachi NX2OOO system. The cross-sectional images of the HfO_2_ thin films were examined by a field emission high-resolution transmission electron microscopy (HR-TEM, JEM-2100F, USA).

## Results and Discussion

**Fig. 1:**
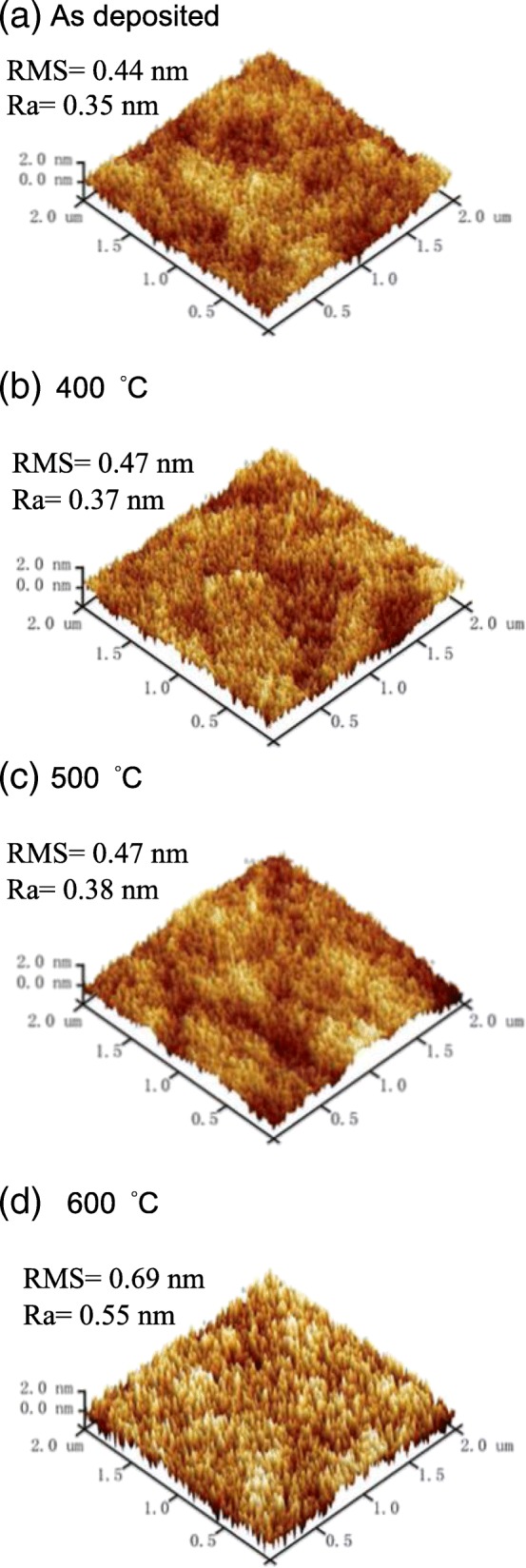
AFM images of **a** as-deposited, **b** 400 °C-annealed, **c** 500 °C-annealed, and **d** 600 °C-annealed HfO_2_ films

Figure 1 shows the AFM images for the HfO_2_ films annealed at different temperatures. The root-mean-square (RMS) and average surface roughness (Ra) values are shown for indicating the surface roughness. The RMS value is 0.44 nm for the as-deposited film. It slightly increases to 0.47 nm when the annealing temperature rises to 500 °C. Further increasing the annealing temperature to 600 °C leads to a significant enhancement in surface roughness with a RMS increasing to 0.69 nm. Same tendency is observed in Ra values. The increase in surface roughness for the annealed films might infer a structural change.

**Fig. 2:**
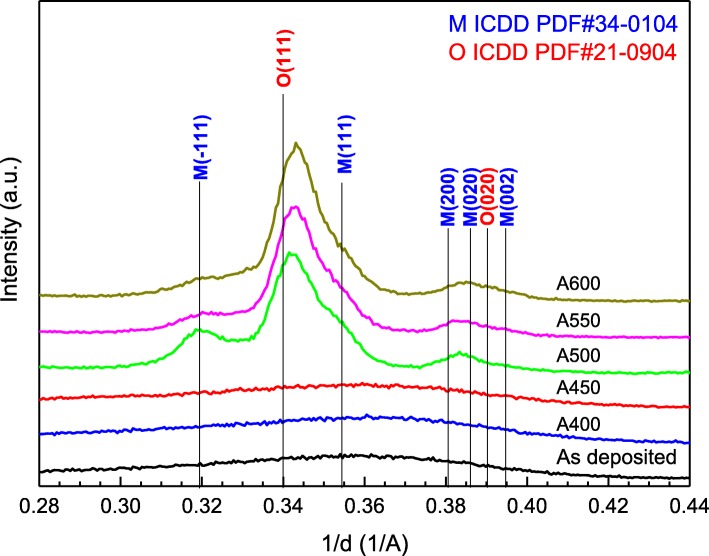
GIXRD spectra of HfO_2_ thin films annealed at different temperatures

Figure 2 shows the temperature-dependent GIXRD spectra of various HfO_2_ thin films. The as-deposited HfO_2_ films is amorphous and remains amorphous after annealing at 400 and 450 °C. At an annealing temperature higher than 500 °C, diffraction peaks appear, indicating the formation of crystalline HfO_2_. The peaks at 1/d = 0.319 and 0.354 Å^−1^ correspond to the − 111 and 111 planes to the monoclinic phase (ICDD PDF#34-0104, space group P21/c), respectively. The peak at 1/d = 0.340 Å^−1^ corresponds to the (111) plane of the orthorhombic phase (ICDD PDF#21-0904, space group Pbcm). Other peaks near 1/d = 0.380~0.395 are the 200, 020, and 002 planes of the monoclinic and the 020 plane of the orthorhombic phases. The results also reveal that the monoclinic phase decrease and the orthorhombic phases increase with the annealing temperature. The orthorhombic HfO_2_ dominates the crystalline structure at higher annealing temperatures. However, the diffraction peaks of orthorhombic HfO_2_ were observed at a lower 1/d (a smaller d-spacing) as compared to that in the ICDD PDF#21-0904. In addition, the shift of 1/d = 0.340 Å^−1^ towards a higher value indicates that the d-spacing decreases with the annealing temperature.

**Fig. 3:**
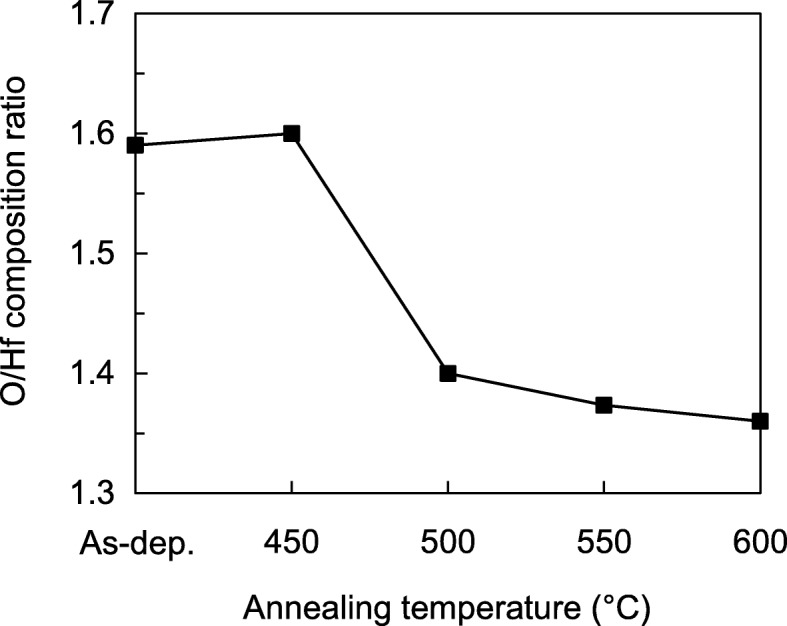
Atomic ratio of oxygen to hafnium for HfO_2_ thin films annealed at different temperatures

The concentrations of Hf and O within the HfO_2_ films were measured using depth profiled XPS. Figure 3 shows the O/Hf composition ratio of the as-deposited and post-annealed HfO_2_ films. The O/Hf ratio decreases from 1.60 to 1.29 with the annealing temperature. Due to the use of N_2_ during the annealing, the HfO_2_ becomes oxygen deficient with the temperature. The oxygen deficient HfO_2_ film also results a smaller d-spacing as mentioned previously.

**Fig. 4:**
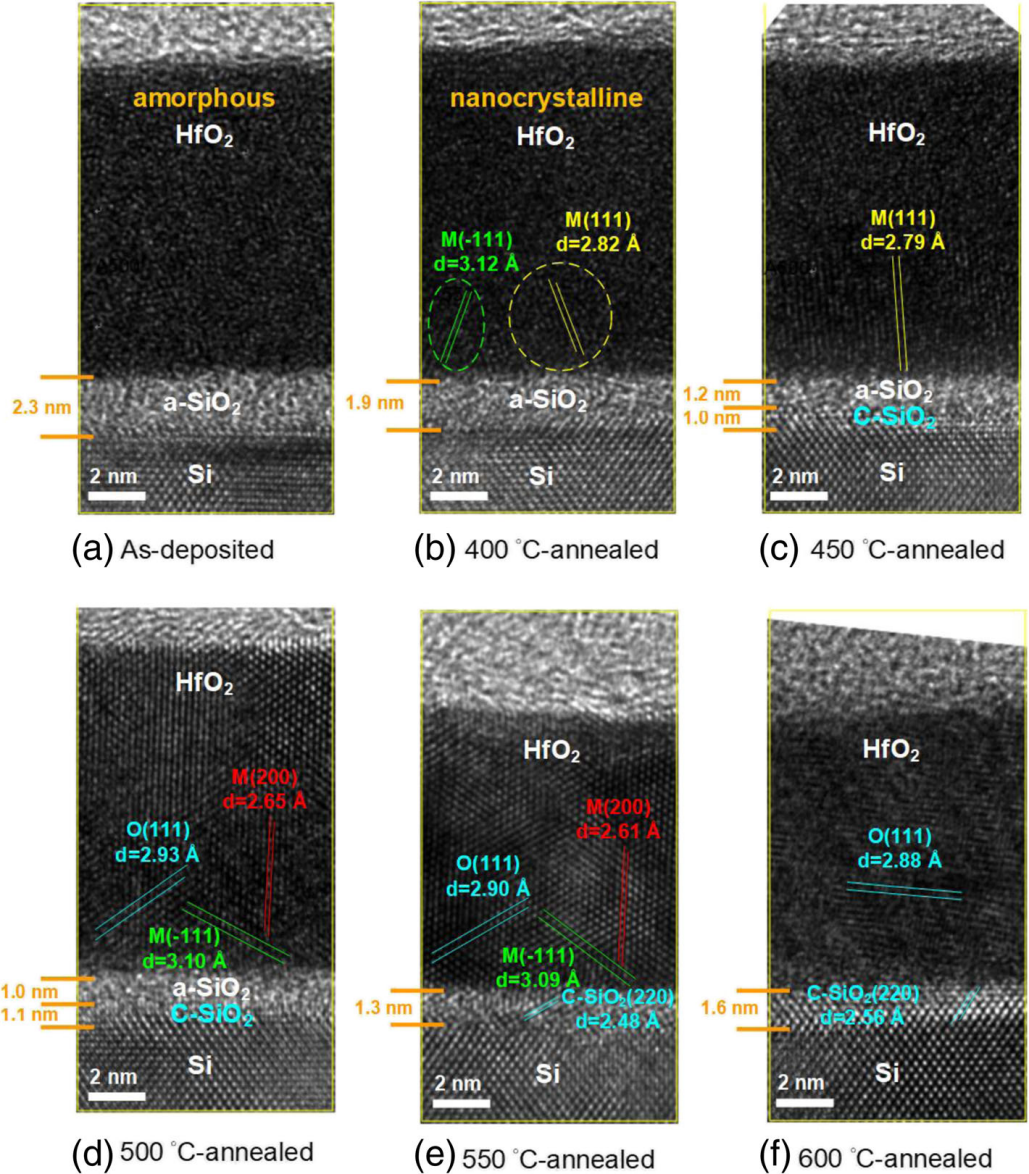
Cross-sectional TEM images of **a** as-deposited, **b** 400 °C-annealed, **c** 450 °C-annealed, **d** 500 °C-annealed, **e** 550 °C-annealed, and **f** 600 °C-annealed HfO_2_/Si

Figure 4a, b, c, d, e, and f show the high-resolution cross-sectional HR-TEM images of as-deposited 400 °C-, 450 °C-, 500 °C-, 550 °C-, and 600 °C-annealed HfO_2_ thin films on Si substrates, respectively. It can be seen that the HfO_2_ layer and Si substrate are clearly exhibited in these images. Additionally, a thin layer with the thickness of 1–2 nm between HfO_2_ and Si substrate could be the SiO_2_ film. As shown in Fig. 4a, there is no obvious lattice arrangement in the as-deposited HfO_2_ film, indicating that this film is amorphous. After annealing at 400 °C, although most regions of HfO_2_ film are still amorphous, we can observe that a fraction of lattice arrangements with the d-spacing values of 2.82 and 3.12 Å are formed in this film. These two d-spacing values are indexed to monoclinic HfO_2_ (111) and monoclinic HfO_2_ (− 111) planes, respectively, and the 400 °C-annealed film shows the nanocrystalline structure. With increasing the annealing temperature from 400 to 600 °C, the crystal quality of HfO_2_ film is gradually enhanced. When the HfO_2_ film is annealed at 500–550 °C, the main lattice arrangements consisting of monoclinic HfO_2_ (− 111), monoclinic HfO_2_ (200), and orthorhombic HfO_2_ (111) can be identified. However, further increasing the annealing temperature to 600 °C, the lattice structure of orthorhombic HfO_2_ (111) still exists in the film, and the other two lattice arrangements gradually disappear. On the other hand, the d-spacing values of orthorhombic HfO_2_ (111) planes for the 500 °C-, 550 °C- and 600 °C-annealed HfO_2_ films are determined to be 2.93, 2.90, and 2.88 Å, respectively. This agrees well with the XRD result that the orthorhombic HfO_2_ (111) diffraction peak shifts towards to the high angle direction with increasing the annealing temperature from 500 to 600 °C. The result reveals that the oxygen content of HfO_2_ film reduces gradually as the annealing temperature is increased. The other interesting phenomenon can be found in the changes of crystal structure and thickness of the SiO_2_ layer. At the as-deposited state, the SiO_2_ layer is amorphous. Even if the sample is annealed at 400 °C, the thermal energy is not high enough to transform the structure of SiO_2_ layer from amorphous to crystalline. Nevertheless, by increasing the annealing temperature from 450 to 600 °C, the crystalline SiO_2_ layer (with the cubic SiO_2_ (220) structure) is formed and its thickness increases from 1.0 to 1.6 nm. It can be observed that the amorphous SiO_2_ layer completely transforms to cubic SiO_2_ structure after annealing the sample at 600 °C. With an increment of annealing temperature from 550 to 600 °C, the d-spacing value of cubic SiO_2_ (220) increases from 2.48 to 2.56 Å. This means that the oxygen content of SiO_2_ layer increases by increasing the annealing temperature. It can be reasonably speculated that the addition of oxygen content in the SiO_2_ layer is attributed to the diffusion of oxygen atoms sourced from the HfO_2_ film. Moreover, the overall thickness decreases for the annealing temperature of 550 and 600 °C and might be related to the increase of the film density caused by crystallization and hydrogen removal.

**Fig. 5:**
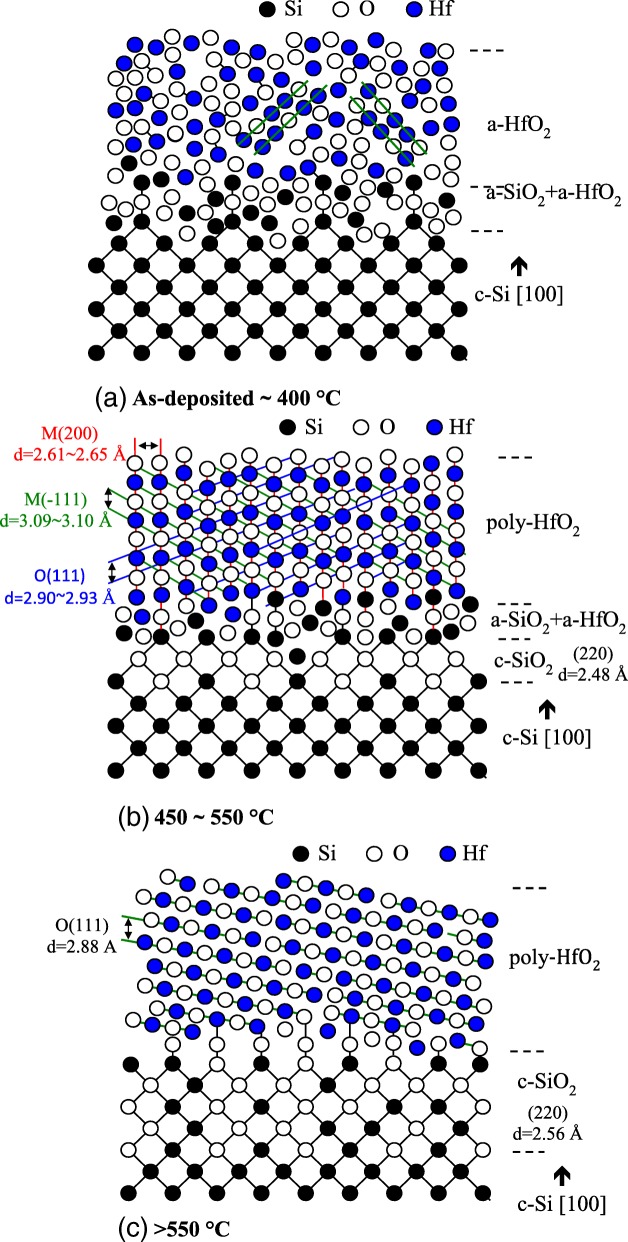
Diagrams of mechanism of crystallization of HfO_2_ films and interfacial layer in the temperature ranges **a** as-deposited to 400 °C, **b** 450 to 550 °C, and **c** beyond 550 °C. The d-spacing value and crystalline orientation are also indicated

Based on the above results, Fig. 5 illustrates the mechanisms of the HfO_2_ films with different annealing temperatures. Considering the annealing temperature is smaller than 400 °C (Fig. 5a), the film is amorphous where Hf and O atoms are randomly arranged. The interfacial layer between HfO_2_ and c-Si wafer is a mixed oxide consisting of a-SiO_2_ and a-HfO_2_. At an annealing temperature of 450–550 °C (Fig. 5b), the HfO_2_ film receives thermal energy leading to a structural change from amorphous to polycrystalline with monoclinic and orthorhombic phases. The crystalline orientation and d-spacing are indicated according to the HR-TEM and GIXRD results. A crystalline SiO_2_ layer is formed. Several works reported an ordered silicon oxide layer at the interface of a-SiO_2_ and (100) c-Si, but the mechanism and atomic-scale structure have remained controversial. Silicon thermal oxidation could be regarded as sequential inserting operations of oxygen atoms into Si-Si bonds, and this induces a large accumulation of compressive strains in the oxidized regions and might possibly cause a structural transformation into ordered oxide at the SiO_2_/c-Si interface [24]. It has also been reported that crystalline oxygen-containing phase could be formed under conditions of high oxygen oversaturation of Si [25] or low interface defect density [26]. From the XPS and TEM images in this work, the HfO_2_ layer is oxygen deficient. The significant amounts of oxygen diffuse from HfO_2_ towards silicon substrate, and this might lead to oversaturation of oxygen at the c-Si interface and formation of crystalline SiO_2_. In this temperature range, the crystalline SiO_2_ layer thickness would increase but the a-HfO_2_ + a-SiO_2_ mixed layer thickness decreases with increasing annealing temperature. At an annealing temperature higher than 550 °C (Fig. 5c), the HfO_2_ structure is dominated by polycrystalline orthorhombic (111) single phase. The interfacial layer is entirely governed by crystalline SiO_2_. The d-spacing decreases for orthorhombic HfO_2_ layer and increases for c-SiO_2_. Although annealing of HfO_2_ is necessary for achieving high Si wafer passivation and dielectric constant, at high temperatures, the resultant crystallization of the HfO_2_ and the interfacial SiO_2_ may reduce the film properties. The annealing temperature of 500 °C is found to obtain the best dielectric constant of 17.2. Further increasing the annealing temperature leads to a reduction in dielectric constant, possibly due to the change in the crystalline phase. Tomida et al. reported that the dielectric constant of HfO_2_ decreases when the structure transformed from polycrystalline to monoclinic single phase [27]. The best passivation of HfO_2_/Si can also be obtained at the annealing temperature of 500 °C, as higher temperatures might lead to a complete c-SiO_2_ interfacial layer and dehydrogenation at the interface.

## Conclusion

HfO_2_ films are prepared using RP-ALD, and effect of annealing temperature on crystalline structure of the HfO_2_ has been investigated. For as-deposited HfO_2_ and that annealed below 400 °C, the HfO_2_ and the interfacial layer are amorphous. With increasing annealing temperature, the d-spacing of orthorhombic reduces while that of the c-SiO_2_ interfacial layer increases, indicating the oxygen diffusion from HfO_2_ to Si interface. Annealing temperature higher than 550 °C shows a HfO_2_ layer with polycrystalline orthorhombic single-phase, and the interfacial layer completely transforms to c-SiO_2_. Although annealing is required for HfO_2_ in many applications such as achieving high passivation of Si wafers and high dielectric constant, the crystallization could be harmful to the film properties. The annealing temperature of 500 °C can have the best Si wafer passivation quality and dielectric constant.

## Abbreviations

AFM: Atomic force microscopy
a-HfO_2_: Amorphous hafnium oxide
ALD: Atomic layer deposition
a-SiO_2_: Amorphous silicon dioxide
c-SiO_2_: Crystalline silicon dioxide
GIXRD: Grazing incident X-ray diffraction
HfO_2_: Hafnium oxide
HR-TEM: High-resolution transmission electron microscopy
N_2_: Nitrogen
O_2_: Oxygen
RMS: Root-mean-square
RP-ALD: Remote plasma atomic layer deposition
RTA: Rapid thermal annealing
TEMAH: Tetrakis (ethylmethylamino) hafnium
XPS: X-ray photoelectron spectroscopy
